# Evaluation of SrAl_2_O_4_:Eu, Dy phosphor for potential applications in thermoluminescent dosimetry

**DOI:** 10.1002/acm2.13251

**Published:** 2021-05-05

**Authors:** Xueyan Tang, Eric D. Ehler, Eric Brost, Damien C. Mathew

**Affiliations:** ^1^ Department of Radiation Oncology University of Minnesota Minneapolis MN USA; ^2^ Department of Radiation Oncology Mayo Clinic Rochester MN USA

**Keywords:** dose response, quality assurance, strontium aluminate, thermoluminescent dosimetry, trapping parameters

## Abstract

**Purpose:**

To evaluate the use of commercial‐grade strontium aluminate phosphorescent powder as a thermoluminescent (TL) dosimeter for clinical radiotherapy beams.

**Materials and Method:**

Commercially available Eu^2+^, Dy^3+^ co‐doped strontium aluminate powder (SrAl_2_O_4_:Eu, Dy) was annealed and then irradiated using 20 × 20 cm^2^ field size, with 6‐MV (PDD_10_ = 70.7) and 18‐MV (PDD_10_ = 79.4) photon beams and and 9‐MeV (R_50_ = 3.6), 15 MeV (R_50_ = 5.9) and 18‐MeV (R_50_ = 7.2) electron beams. To calibrate the relationship between the TL readings and the irradiated doses, TL glow curves were acquired for doses up to 600 cGy at all beam energies. For the percentage depth dose (PDD) measurement, the SrAl_2_O_4_:Eu, Dy powder was sandwiched by solid water phantoms, with varying thickness of solid water placed above to determine the depth. PDDs were measured at four representative depths and compared against the commissioning depth dose data for each beam energy.

**Results:**

Linear dose response models of doses up to 200 cGy were created for all beam energies. Superlinearity was observed with doses greater than 200 cGy. The PDD measurement acquired experimentally agrees well with the commissioning data of the medical linear accelerator. Trapping parameters such as order of kinetics, activation energy and frequency factor have been obtained via TL glow curve analysis.

**Conclusion:**

The linear dose response demonstrates that SrAl_2_O_4_:Eu, Dy is a potential TLD dosimeter for both electron beams and photon beams at different beam energies. The PDD measurements further support its potential use in quality assurance and radiation dosimetry.

## 1 INTRODUCTION

Strontium aluminate phosphors have been widely used in industry as a result of their high phosphorescence intensity and long persistence of phosphorescence.^1^ The prolonged light emission is due to the transitions of the charged carriers within the crystal into and out of metastable levels, following the initial absorption of energy from an external source.^2^ Using rare earth ions as co‐dopants, new trapping levels with appropriate depths for electron releasing at room temperature are formed, and thus, their phosphorescence properties are improved.^3^ Strontium aluminate co‐doped with Eu^2+^ and Dy^3+^ ions (SrAl_2_O_4_:Eu, Dy) is one of the most popular phosphors because of its very intense and long lasting afterglow.^4^, ^5^

When a persistent phosphor is excited by ionizing radiation at room temperature, part of the absorbed energy converts to immediate luminescence and persistent afterglow (shallow traps and intermediate traps), while the rest is stored and accumulated in deeper traps that require extra stimulation such as heating to release.^2^ The immediate luminescence and afterglow can be observed at room temperature. However, to investigate the energy stored in deep traps in materials, stimulation in the form of heat (thermoluminescence) or light (optically‐stimulated luminescence) is required to liberate the charged carriers.^6^

For a material to qualify as a thermoluminescent dosimeter (TLD), high electron‐hole pair creation and trapping efficiency is required.^7^ In addition, the time integration of the TL signal obtained during thermal stimulation should be proportional to the delivered dose. Several commercial TLDs have been thriving for decades such as calcium fluoride and lithium fluoride.^8^ However, these popular TLDs have their own limitations. For example, the hazards identification of calcium/lithium fluoride compound is marked as skin corrosion/irritation category 2, which forbids it to have direct contact with patients and limit its application form in in vivo dosimetry.^9^ The TL dosimeters are relatively economical compared to other dosimeters such as films because of their reusability. Even so, it still costs more than $10/gram for the LiF TLD powder. The exploration of lower toxicity, more cost‐efficient and user‐friendly TLD materials that have comparable accuracy is still of great interest.

The incorporation of the rare earth ions into the phosphor material leads to dense trapping levels suitable for TL purpose.^7^, ^10^ The effective atomic number of strontium aluminate family is quite high compared to the tissue‐equivalent TLDs, making it possibly suitable for high radiation dosimetry.^11^ In the past few years, the dose response of certain strontium aluminate phosphors for X‐ray and Co‐60 beams have been calibrated via thermoluminescence.^12^, ^13^, ^14^ These studies have found a linear dose response relationship in the kGy range, which is much greater than the doses of concern for in vivo dosimetry in radiation therapy.

The purpose of this study is to investigate the thermoluminescence properties of irradiated SrAl_2_O_4_:Eu, Dy phosphor to explore its use in radiation dosimetry, for both electron and photon beams within the in vivo dosimetry dose range. The SrAl_2_O_4_:Eu, Dy phosphor is widely used as a glow in the dark pigment and can be directly painted onto the skin without side effects. It is a great candidate for TL dosimeter not only because of its nontoxicity and $0.3/gram price but also because of its real‐time phosphorescent emission, which can potentially offer real‐time fluorescence monitoring and will be studied in subsequent studies. Dose response experiments are performed using clinically commissioned electron and photon beams of different beam energies. Percentage depth dose (PDD) is measured to verify its potential use in quality assurance (QA) routines. To the best of our knowledge, the trapping parameters such as order of kinetics, activation energy, and frequency factor of the SrAl_2_O_4_:Eu, Dy phosphor have been calculated for the first time to get a better understanding of its phosphorescence mechanism.

## 2 MATERIALS AND METHODS

### 2.A Sample preparation

Commercial SrAl_2_O_4_:Eu, Dy powder (Techno Glow Products, Ennis, TX, USA) was annealed using a TLD annealing furnace (Radiation Products Design, model 168‐001) at 400°C for 24 hours to release TL energy traps in the materials. After that, the powder was divided equally for dose calibration and PDD measurements for 6‐MV (PDD_10_ = 70.7) and 18‐MV (PDD_10_ = 79.4) photon beams and 9‐MeV (R_50_ = 3.6), 15‐MeV (R_50_ = 5.9), and 18‐MeV (R_50_ = 7.2) electron beams. Measured amounts of SrAl_2_O_4_:Eu, Dy powder was sealed inside flat transparent Polypropylene bags. The bags were then placed inside ~1.5 × 1.5‐inch envelopes made from the light‐tight paper from previously used film envelopes and sealed using darkroom tape (3 M Scotch Model 235, Saint Paul, MN, US).

### 2.B Irradiation process

**Fig. 1:**
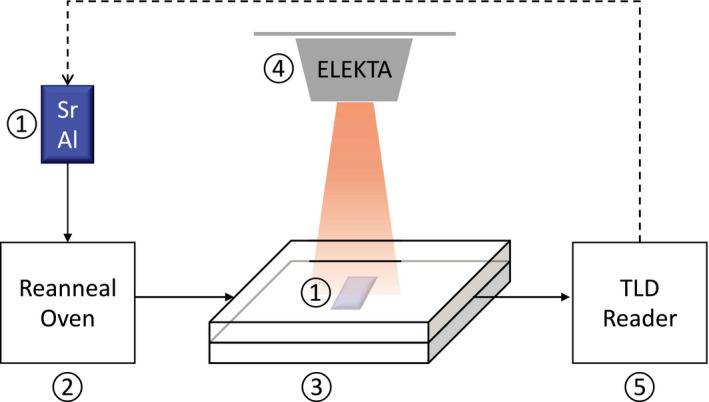
Schematic of the experiment workflow. ① SrAl_2_O_4_:Eu, Dy powder sealed inside flat Polypropylene bags, ② Reanneal Oven (Radiation Products Design, model 168‐001), ③ Solid water phantoms of different thickness, ④ Elekta Synergy (Stockholm, Sweden), and ⑤ Harshaw TLD reader (Model 3500, Waltham, MA).

A 5‐cm thick slab of 30 × 30 cm^2^ solid water was placed with its center aligned to the central axis of the Elekta Synergy (Stockholm, Sweden) linear accelerator. Superflab bolus (Eckert & Ziegler, model 8117‐0.2) was placed to the top side of this slab. Sample bags were then placed on top of the bolus and sandwiched by additional solid water slabs with varying thickness (Fig. 1).

#### 2.B.1 Dose calibration curve

For the dose calibration measurements, *d*_max_ depth was set for each beam with an SSD of 100 cm for convenient dose conversion from monitor units to cGy. The *d*_max_ value for each beam is shown in Table 1. SrAl_2_O_4_:Eu, Dy powder was irradiated at 20 × 20 cm^2^ field size to ensure that the powder was inside the uniform portion of the field. Monitor units of 20, 40, 100, 200, 400, and 600 were used to irradiate individual samples on the Elekta Synergy. Six trials per subgroup were measured to provide statistical error. The delivered doses were compared against the commissioning data for the Elekta. The outputs of the Elekta were measured monthly during the machine QA and agree with the commissioned data within 1.5%.

**Table 1:** *d*_max_ for each beam quality.

Beam quality	Photon		Electron		
	6 MV	18 MV	9 MeV	15 MeV	18 MeV
*d*_max_ (cm)	1.7	3.5	2.2	3.0	4.0

#### 2.B.2 PDD measurement

For the PDD measurements, three additional depths along the commissioned PDD curves were explored besides *d*_max_ for each beam energy (Table 2). SrAl_2_O_4_:Eu, Dy powder was irradiated at 20 × 20 cm^2^ field size with a fixed monitor unit of 200 using the Elekta Synergy. The measurements were then compared against the commissioned PDD curves for the Elekta.

**Table 2:** Depths of the percentage depth dose measurement for each beam quality.

Beam quality	Photon		Electron		
	6 MV	18 MV	9 MeV	15 MeV	18 MeV
Depth 1 (cm)	1.7	3.5	1.0	1.0	1.0
Depth 2 (cm)	5.0	5.0	2.2	3.0	4.0
Depth 3 (cm)	10.0	10.0	3.0	5.0	6.5
Depth 4 (cm)	15.0	15.0	3.5	6.0	7.2
Depth 5 (cm)	NA	NA	4	NA	NA

### 2.C Read out

The SrAl_2_O_4_:Eu, Dy samples were stored in a dark room for more than 24‐hours post‐irradiation to allow the non‐TL light emission to decay. A Harshaw TLD reader (Model 3500, Waltham, MA) was used to read out the dose response of the samples. A linear temperature ramp from 20°C to 350°C with a high temperature hold for 60 seconds was applied. When the reading is finished, the SrAl_2_O_4_:Eu, Dy powder can be recycled and re‐annealed to be used again (Fig. 1).

## 3 RESULTS

### 3.A Calibration curves of the dose response

**Fig. 2:**
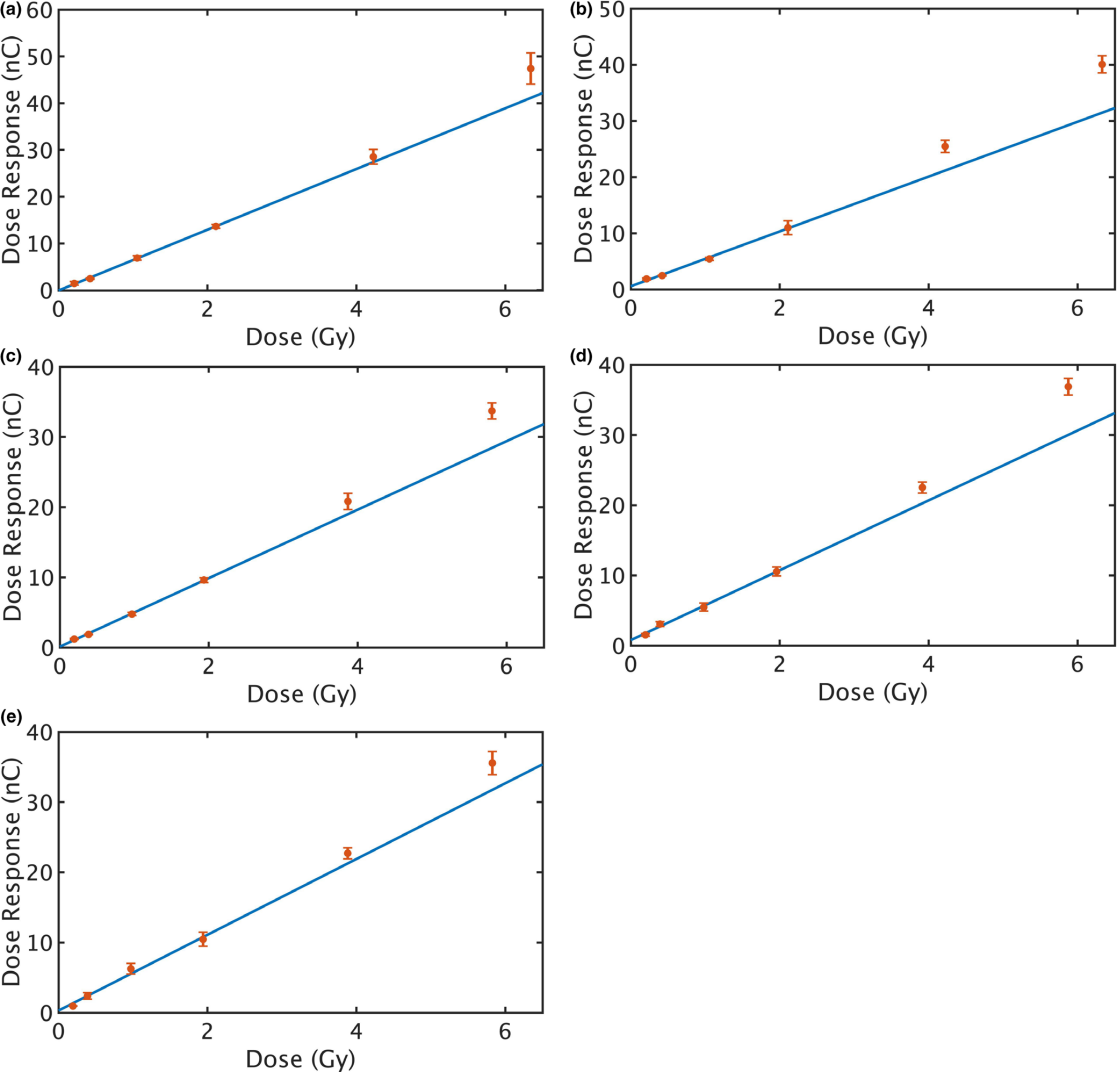
Linear regression models of the dose response as a function of the delivered dose up to 200 cGy, for SrAl_2_O_4_:Eu, Dy samples irradiated with different beam qualities: (a) 6‐MV photon beam, (b) 18‐MV photon beam, (c) 9‐MeV electron beam, (d) 15‐MeV electron beam, (e) 18‐MeV electron beam. Red markers are the integrated TLD readings averaged among six trials, with error bars showing the standard deviation. The blue lines are the linear fits of the experiment data up to 200 cGy.

TL glow curves were measured for all SrAl_2_O_4_:Eu, Dy samples. The TL signal was integrated across the entire temperature ramp in each glow curve to obtain the dose response. After irradiation, the results showed a strong linear relationship between the dose response and the delivered dose up to 200 cGy for all beam energies; superlinearity was observed above 200 cGy (Fig. 2), about the same region as for the LiF dosimeter.^15^ For all the plots in Fig. 2, X‐axis is the delivered dose, and *Y*‐axis is the dose response. The red dots are the integrated TLD readings averaged among six trials, with error bars showing the standard deviation. The blue lines are the linear fits of the experiment data up to 200 cGy. The *R* ^2^ values for all the linear regression models are above 99.5% and the linear fits are given as below:

(1) $$6\;\text{Mv}\;R_{\text{TLD}}\;=\;6.49\left(\pm 0.39\right)\times D‐0.05\left(\pm 0.47\right),$$

(2) $$18\;\text{Mv}\;R_{\text{TLD}}\;=\;4.88\left(\pm 0.61\right)\times D+0.55\left(\pm 0.74\right),$$

(3) $$9\text{Mev}\;R_{\text{TLD}}\;=\;4.88\left(\pm 0.33\right)\times D+0.13\left(\pm 0.37\right),$$

(4) $$15\;\text{MeV}\;R_{\text{TLD}}\;=\;4.98\left(\pm 0.68\right)\times D+0.78\left(\pm 0.76\right),$$

(5) $$18\;\text{MeV}\;R_{\text{TLD}}\;=\;5.4\left(\pm 1.34\right)\times D+0.30\left(\pm 1.49\right),$$

where R_TLD_ is the integrated TLD reading in nC, D is the delivered dose in Gy, uncertainties of the fitted parameters are calculated for 95% confidence interval. The coefficients of the linear regression models are quite similar for different beam energies, with slightly better sensitivity for proton beams. Because the parameters are highly dependent on the sample qualities, it is recommended to calibrate each batch of samples before use.

### 3.B PDD measurement

**Fig. 3:**
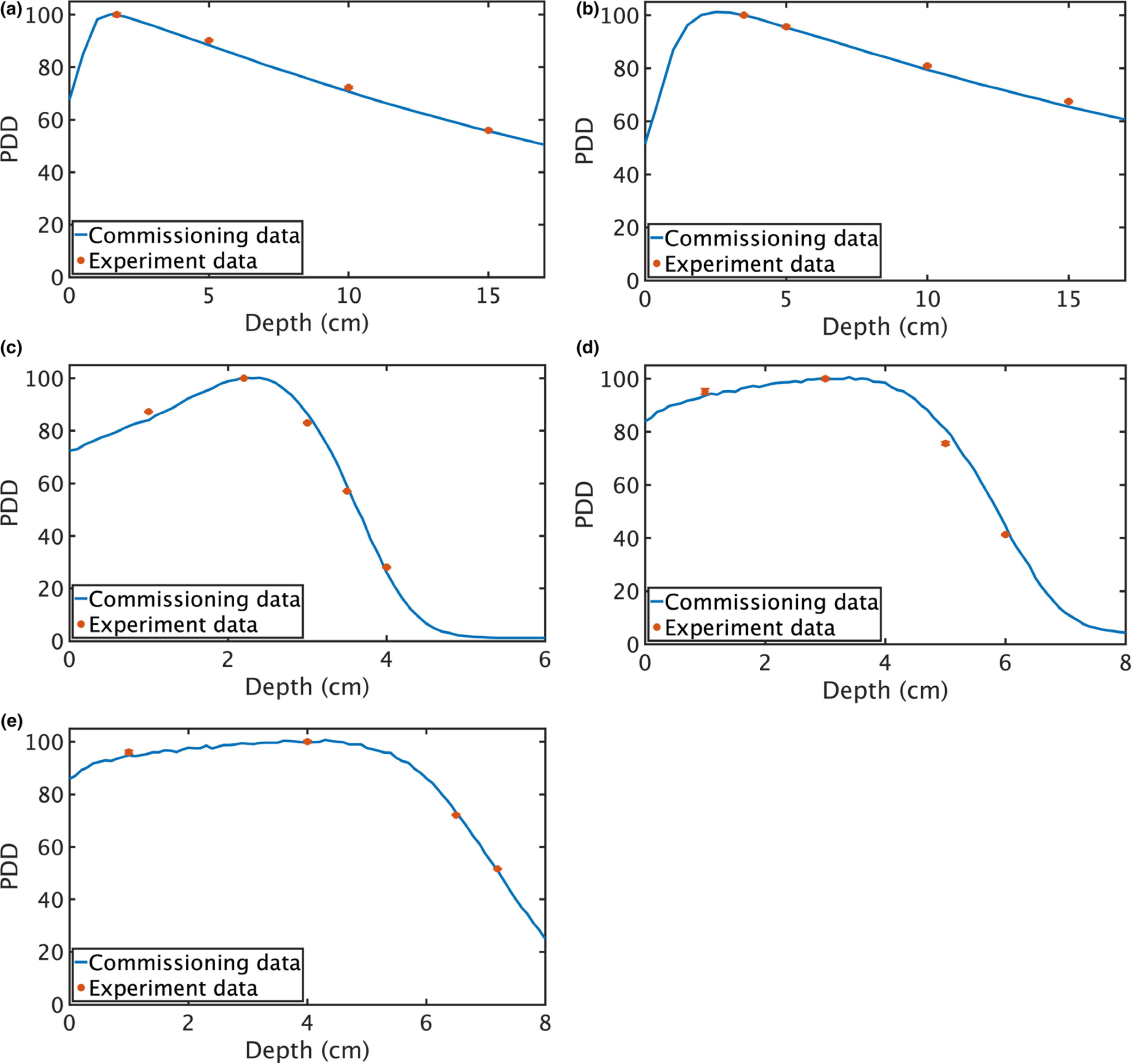
PDD measured using SrAl_2_O_4_:Eu, Dy for different beam qualities, overlapped with the commissioning data of Elekta Synergy: (a) 6‐MV photon beam, (b) 18‐MV photon beam, (c) 9‐MeV electron beam, (d) 15‐MeV electron beam, and (e) 18‐MeV electron beam. The vertical error bars account for the standard deviation among the six trials of one subgroup.

The PDDs at several representative depths (Table 2) were measured for each beam quality. All the experimentally obtained PDDs agree with the commissioning data of the Elekta Synergy, with most of the subgroups having less than 5% standard deviation among the six trials (Fig. 3). The experimentally acquired PDDs for 6‐MV photon beam at 5, 10, and 15 cm are 90.2, 72.3, and 56.0, while the PDDs from the commissioning data are 88.4, 70.7, and 55.7. The measured PDDs for 18‐MV photon beam at 5, 10, and 15 cm are 95.7, 80.9, and 67.5, while those from the commissioning data are 95.4, 79.4, and 65.5. All the measured data agree with the commissioning data within 2%.

Due to the high gradient in the electron beams, a comparison of R_50_ (half‐value depth in water) would likely be more prudent.^16^ Using linear interpolation of the experimentally measured data, the R_50_ of 9 ‐, 15‐, and 18‐MeV electron beams are calculated to be 3.62, 5.79, and 7.04 cm, respectively; while those from the commissioning data are 3.6, 5.9, and 7.2 cm.

### 3.C Trapping parameters calculation

Since trapping parameters are dominated by the properties of the trapping centers, the determination of the trapping parameters using the glow curves is helpful for understanding the nature of the SrAl_2_O_4_:Eu, Dy phosphor.^7^ From measurements, we found that even at different dose levels, with different beam qualities, the glow curves had the same peak shape. This is expected because the photon beams are indirectly ionizing radiation; it is the induced secondary beta particles that excite the sample.^16^ As a result, despite different beam types and energies, when it comes to the total energy directed into the SrAl_2_O_4_:Eu, Dy samples from the radiation, photon beams and electron beams share the similar physical process. We calculated the trapping parameters based on the glow curve measured at the dose level of 600 cGy using 18‐MeV electron beams.

**Fig. 4:**
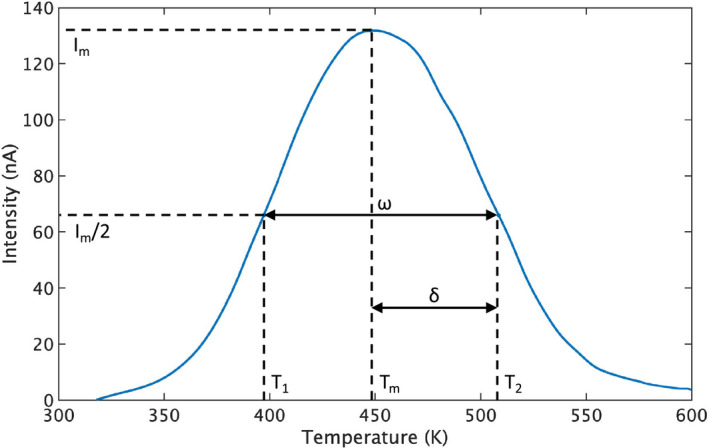
A glow curve of the SrAl_2_O_4_:Eu, Dy phosphor showing the parameters of δ = T_2_ − T_m_ and ω = T_2_ – T_1_ at 600‐cGy dose level using 18‐MeV electron beam.

The calculation of the trapping parameters starts with calculating the symmetry factor µ_g_:

(6) $$\mu_{g}\;=\;\frac{\delta }{\omega },$$

where δ is the temperature difference between the higher half intensity temperature T_2_ and the temperature of the glow maximum T_m_, δ = T_2_−T_m_, ω is the full width at half maximum that is calculated from the temperature difference between T_2_ and the lower half intensity temperatureT_1_, ω = T_2_−T_1_.^6^ All the temperature units are in Kelvin (K). From the glow curve (Fig. 4), δ is calculated to be 60.4 K, while ω is 111.4 K, which gives a µ_g_ of 0.54. A second order peak is characterized by µ_g_ = 0.52.^17^ Thus, a µ_g_ of 0.54 is close enough for us to conclude that it is second order kinetics.

Assume that the frequency factor is independent of the temperature, the activation energy for a second‐order peak can be calculated based on the measurement of T_m_ and ω:

(7) $$E_{\omega }=3.54\times \left({\text{kT}}_{m}^{2}/\omega \right)‐2{\text{kT}}_{m},$$

where k is the Boltzmann constant in eV/K.^17^ With a T_m_ of 448.1 K and a ω of 111.4 K, the activation energy equals 0.47 eV.

The frequency factor s is also defined as the “attempt‐to‐escape frequency,” which is calculated from the activation energy:

(8) $$\beta E/{\text{kT}}_{m}^{2}\;=\;{\text{se}}^{‐E/{\text{kT}}_{m}},$$

Where β is the heating rate and E is the activation energy.^17^ At a heating rate of 25 K/s, the frequency factor of the SrAl_2_O_4_: Eu, Dy phosphor equals 1.3 × 10^5^/s.

## 4 DISCUSSION AND CONCLUSIONS

Thermoluminescence properties of the SrAl_2_O_4_:Eu, Dy phosphor irradiated by different beam qualities have been presented in this work. The linear relationship between the TL‐dose response and the delivered dose shows that SrAl_2_O_4_:Eu, Dy is a potential TL dosimeter for both photon and electron beams in the clinical dose range. Superlinearity is observed above 200 cGy, which makes its dynamic range comparable to the other common TL dosimeters and wide enough for the application in QA and in vivo dosimetry.^15^, ^18^ Previous studies investigating the TL response to radiation of strontium aluminate had different dopants (SrAl_2_O_4_:Eu) or different chemical composition (SrAL_4_O_7_:Dy); the studies focused on doses of 295–2360, 50–200, and 50–2360 Gy, which were beyond the scale of doses measured for in vivo dosimetry in radiation oncology.^12^, ^13^, ^14^ Compared to the existing studies, this work covers much wider beam quality range as well as more appropriate dose range for in vivo dosimetry, with more focus on the clinical application of the SrAl_2_O_4_:Eu, Dy phosphor.

The PDD measurement shows that SrAl_2_O_4_:Eu, Dy phosphor can be used in QA routines with relatively high accuracy: less than 5% percentage error among the six trials and less than 2% difference from the commissioning data. The standard deviation of using LiF TL dosimeter is usually about 5%, while the overall accuracy is around 1%.^19^ The accuracy of the SrAl_2_O_4_:Eu, Dy phosphor is comparable to that of the LiF dosimeter.

The calculated trapping parameters show that the glow curve from SrAl_2_O_4_:Eu, Dy phosphor follows second‐order kinetics, similar to that of the Eu doped strontium aluminate and strontium borate.^13^, ^20^ However, the activation energy and frequency factor of the SrAl_2_O_4_:Eu, Dy phosphor are smaller compared to the Eu‐doped compounds, possibly as a result of incorporating the Dy dopants. Different time temperature profiles for the TLD study are planned to be used in future experiments to explore the effects on the trapping parameters.

Compared to the other widely used TL dosimeters, the SrAl_2_O_4_:Eu, Dy phosphor has the advantage of nontoxicity, and as a result, a container is not necessary. The SrAl_2_O_4_:Eu, Dy powder can be readily incorporated into other materials such as gels and plastic, which could be molded to conform to a patient's or a phantom's contour. Its price is also 30 times lower than the popular LiF TLD powder, making it more suitable for daily clinical use. Even though it is not tissue‐equivalent, the correction of a reading to the dose in tissue can be made as long as the radiation quality is known.^21^ The phosphorescence property of the SrAl_2_O_4_:Eu, Dy phosphor can be potentially utilized to visualize real‐time spatial fluence during radiation therapy besides the TL dose response.

Future work will focus on further improving the dosimetry accuracy of the SrAl_2_O_4_:Eu, Dy material such as shaping the powder into chip form, optimizing the reanneal procedure and optimizing the time‐temperature profile used for the read‐out process. We also plan to explore the phosphorescent properties of SrAl_2_O_4_:Eu, Dy when irradiated with clinical radiotherapy beams. This could lead to a 2D real‐time fluence map using SrAl_2_O_4_:Eu, Dy coated phantoms and in vivo dosimetry devices.

## CONFLICT OF INTEREST

No conflicts of interest.

## Data Availability

The data that support the findings of this study are available from the corresponding author upon reasonable request.
